# Evaporation driven buckling of a drop laden with graphene oxide nanosheets†

**DOI:** 10.1039/d4sm01342e

**Published:** 2025-02-17

**Authors:** Suriya Prakash, Eva Krolis, Alvaro Marin, Lorenzo Botto

**Affiliations:** a Department of Process & Energy, Faculty of Mechanical Engineering, TU Delft The Netherlands L.botto@tudelft.nl; b Physics of Fluids, Faculty of Science & Technology, University of Twente Enschede The Netherlands

## Abstract

The time-dependent shape of an evaporating spherical water drop containing graphene oxide (GO) nanosheets is measured for varying solid concentration, humidity level, and pH. The drop is sitting on a superhydrophobic surface, depinned from it. Three different stages of evaporation are identified: isotropic retraction of the drop interface, buckling of the shell of particles accumulated at the fluid interface, and shrinking of the buckled shell at constant shell shape. Marked differences between acidic and basic drops are reported. It is argued that this feature is caused by the pH-dependent interfacial adsorption of the GO particles. For intermediate values of GO concentration, dried capsules with remarkably repeatable folding patterns could be obtained, whose mode numbers are compatible with those predicted by an inertialess, linear elastic shell model. When redispersed in water, the dried capsules from acidic drops retain their shape better than capsules from basic drops.

## Abstract 2

Faster evaporation leads to higher buckling modes in dried graphene oxide capsules.

## Graphical abstract

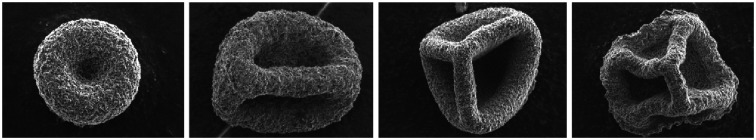

## 1 Introduction

A small spherical drop containing micro or nanoparticles can form a semi-solid (visco-elastic) particle “skin” when the droplet evaporates. The “skin”, or particle shell, is caused by the motion of the contracting fluid interface that sweeps the particles suspended inside the drop.^1,2^ In addition to its fundamental interest, this phenomenon has been studied due to its application to spray drying, for the large-scale production of dried microstructured particles to be used in food products,^3^ lubricants,^4^ pharmaceuticals^5^ and functional materials.^6–9^

The recent explosion of interest in 2D material nanosheets (graphene, graphene oxide, boron nitride, MXenes, MOS_2_, *etc.*) has stimulated research on the use of evaporation driven buckling (“crumpling”) of shells formed from droplets laden with dilute suspensions of these colloidal materials.^10–13^ Graphene oxide (GO), which is easily dispersible in water at dilute concentrations, is the most used 2D material in spray drying applications,^14–16^ but its behavior in evaporating drops is not well understood.

This paper reports a comprehensive investigation of the evaporation of GO-filled droplets and of the different stages that characterize the shell buckling process. We work with single millimeter-sized droplets that are placed on a superhydrophobic surface. In this configuration a colloidal droplet adopts a spherical shape during evaporation until buckling occurs. This configuration has previously been used to investigate evaporating particle-laden droplets.^17–19^ As far as we are aware, the current study is the first report of repeatable measurements of the time-dependent single GO drop evaporation under controlled conditions of humidity, GO concentration, and pH.

Only very few experimental studies on evaporation of GO droplets are available.^14,15,20–22^ These studies have provided a useful characterization of the shapes of dried capsules formed from spray drying, but have not provided insights into the drying process at the level of a single drop. Spray drying of GO–water suspension has been reported to produce dried capsules whose shape are highly polydispersed, and dependent on the flow conditions (temperature, humidity level, flow rate). A relevant study on GO reports dissolution of an aqueous GO solution deposited on a hydrophobic substrate immersed in the liquid phase of ethanol in toluene.^23^ In this study, the time dependent radius of the drop was measured, and found to follow diffusion-limited evaporation dynamics (square of the drop radius *R* linear in time *t*). In this study, the structures formed were porous and not hollow. Studies on evaporation of drop containing spherical colloids are abundant.^18,19,24–29^ These studies indicate that a particle shell is only obtained for large values of the Péclet number Pe based on the translational diffusivity *D*_p_ of the particles, the initial drop radius *R*_0_ and the characteristic velocity *v*_i_ of the fluid interface (or alternatively based on the ratio of diffusion and evaporation times^1,30,31^). For a given temperature, the interface velocity depends primarily on the humidity level, with low humidity giving fast evaporation because of the large difference in water vapor concentration between the drop surface and regions away from the drop. For slow drying, Brownian diffusion leads to a uniform distribution of particles inside the drop and therefore to dried structures that are not hollow. The thickness of the shell, and therefore whether a hollow capsule or a filled ball is obtained, depends also on the initial particle concentration *ϕ*_0_.^2,32^ Assuming that all the particles accumulate in the shell adjacent to the fluid interface, the shell thickness can be estimated from a basic mass conservation argument.^1^

A possible distinction between GO and other colloidal particles is interfacial adsorption. GO is known to be amphiphilic, *i.e.* it has a tendency to adsorb at fluid interfaces.^33–35^ The degree of amphiphilicity depends on the amount of oxygen groups present at its surface, but adsorption energies much larger than the thermal energy has been reported both in simulation and experiments.^36,37^ Furthermore, the important effect of GO on interfacial rheology of flat interfaces is well recognized.^38^ Adsorption of GO at fluid interfaces is pH dependent.^34,36^ The amphiphilic character of GO may have implications for the onset of buckling, and that is why we include pH in our control variables. When a fluid interface is populated with adsorbed particles, buckling upon interface compression occurs even when a thick particle layer is not present in the region adjacent to the interface *i.e.* in the absence of a shell layer.^39–42^ The possible importance of GO amphiphilicity on interfacial stresses has not been discussed in the context of GO droplet evaporation experiments.

The literature on evaporation-driven buckling, also of spherical particles, has up to this point only focused on the effect of hydrophilic particles, *i.e.* particles that are completely wet by the liquid. Buckling can occur when the compression of the shell formed by particles completely wetted by the liquid is sufficiently large so that the force of repulsion between charge-stabilized particles is overcome and the particle are forced to adhere to each other.^1^ For large repulsive forces, charge-stabilized particles can also form a glassy repulsive state at volume fractions smaller than the packing fraction. If the pressure corresponding to this repulsion is sufficiently large, a buckling instability can occur.^29^ These explanations are plausible and may apply to most cases, but neglect the possible contributions of interfacial particles, which may be important in all situations where non-negligible interfacial adsorption occurs.

The shape of the dried GO buckled structure (capsule) is often of interest, because the morphology of the capsule determines its volumetric surface area and the stability against aggregation of the capsules upon re-dispersion in solution.^14^ The morphology of buckled shells obtained from drying drops containing spherical colloids has been studied experimentally in ref. 1, 26, 30 and 43. These experiment reported the formation of either torus-shaped capsules^26,30,43^ or capsules with multiple depressions.^1,28^ The experiments of ref. 28 with spherical colloids are particularly relevant as the authors could obtain buckled shapes with high repeatability, by a careful control of the initial drop diameter and solid concentration. The buckled shapes were found to depend on the rate of drying. Specifically, the number of depressions were found to increase for increasing evaporation rates.^28^ A possible cause of this correlation was not proposed.

In our experiment, we are able to control the main parameters that govern the evaporation process, namely the initial GO particle concentration *ϕ*_0_, the humidity level RH, and the solution's pH. The use of a camera, by which we can observe the drop from the side, allows us to observe the different stages of evaporation, detect with precision the onset of buckling and quantify the post-buckling morphologies. Scanning electron microscopy allows us to analyze the shape of the dried and buckled GO capsules.

## 2 Experimental methods

**Fig. 1:**
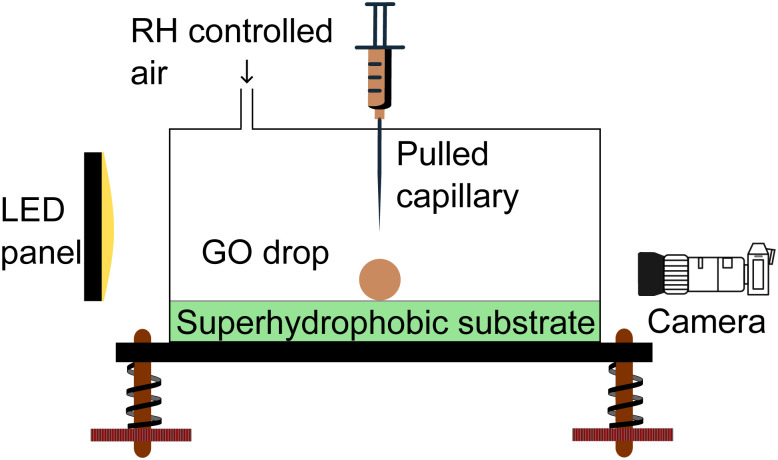
Schematic of the experimental setup.

To study the evaporation of a single GO drop, we place the drop on a superhydrophobic substrate. The superhydrophobic surface is a SiO_2_ substrate with fractal-like micro-structure, coated with fluoroctatrichlorosilane.^19^ The setup is contained in an acrylic chamber equipped with a humidity controller that maintains a set relative humidity RH (Fig. 1).

Due to the superhydrophobicity of the substrate, the drop rolls if the substrate is not perfectly horizontal. Therefore, the humidity chamber with the substrate is mounted on an anti-vibration table on leveling screws. The drop is produced by a syringe with a pulled capillary needle. The syringe is mounted on a precision linear stage platform that is used to gently place the drop on the substrate. We used a 1 ml syringe (B Braun Injekt-F Fine Dosage) housed in a 3D printed screw feeder. The initial drop diameter 2*R*_0_ = 1.5 ± 0.03 mm is smaller than the capillary length, so the drop is approximately spherical.

The drop is observed from the side by a camera equipped with a parfocal zoom lens, with a time resolution of 1 fps. The drop projected area *A* and contact angle are measured by detecting the drop edges with an in-house developed MATLAB code. The drops of pure water and GO suspension are in a Cassie–Baxter state and undergo evaporation at a constant contact angle (*θ* ≈ 155°) for most of the drop's life time (see ESI†).

The graphene oxide suspensions are purchased from Graphenea. The average lateral size of the particles is ℓ = 1.08 ± 0.44 μm measured by scanning electron microscopy (SEM, JEOL JSM 6500 F). The particle thickness is *t* = 1.00 ± 0.14 nm measured by atomic force microscopy (Bruker). See Section II in ESI† for more details on sample preparation and characterization of sheet sizes. The densities of the purchased solutions are measured by a density meter (DMA 5000 Anton Paar). The density of the suspension is *ρ*_GO-sus_ = *ϕρ*_GO_ + (1 − *ϕ*)*ρ*_w_, where *ρ*_GO-sus_, *ρ*_w_ and *ρ*_GO_ are the densities of purchased GO suspension, water and GO sheets, respectively. From this expression, the volume fraction *ϕ* of GO particles is calculated as (*ρ*_GO-sus_ − *ρ*_w_)/(*ρ*_GO_ − *ρ*_w_). The density of water and GO suspension are measured with an accuracy of ±1 kg m^−3^. By assuming that the density of the GO sheets^44^ is ≈2000 ± 100 kg m^−3^, the volume fractions of GO in the suspensions is calculated to be ≈0.0023 ± 0.0002 and ≈0.0034 ± 0.0004 for acidic and basic suspensions, respectively.

The bending energy of the GO sheets used in this study can be estimated to be of the order *Et*^3^ ∼ 2 × 10^−16^ J taking *E* = 200 GPa,^45^ which is much larger than *k*_B_*T* ∼ 4 × 10^−21^ J; here *k*_B_*T* is the thermal energy at standard conditions of temperature. Thus, the sheets do not fold on themselves due to thermal fluctuations. Moreover, it has been shown by *in situ* confocal microscopy^46^ that GO sheets remain flat when suspended in water, and at high concentration they show the nematic order expected for relatively rigid sheets.^47^

The Péclet number, based on the drop drying time *τ*_dry_, is Pe = *R*_0_^2^/(*D*_p_*τ*_dry_),^1^ where *D*_p_ is the translational diffusion coefficient of the GO particles. The particle diffusivity is calculated as *D*_p_ = *k*_B_*T*/(*fμ*ℓ), where *k*_B_ is the Boltzmann constant, *T* is the absolute temperature, *μ* the viscosity of water, and *f* the coefficient of hydrodynamic resistance to translation. Since GO sheets are thin and can be assumed to remain planar in aqueous dispersion at not too high values of the shear rate,^46^ we use for *f* the value 48/7 appropriate for isotropically oriented, infinitely thin disks.^48^ The diffusion coefficient is estimated to be 5.9 × 10^−13^ m^2^/s, leading to a Péclet number in the range ∼150–500 for RH varying between 30% and 80%. The Stokes settling velocity *v*_s_ of the GO particles, which can be estimated with the formula *v*_s_ = π(*ρ*_GO_ − *ρ*_w_)ℓ*tg*/(4*fμ*) ≈ 4.4 nm s^−1^, is much smaller that the air–water interface velocity *v*_i_ ≈ 0.2 μm s^−1^ measured at the start of the evaporation. Since *v*_s_ ≪ *v*_i_, we neglect the effect of gravity on the particle distribution.

The pH of the purchased suspensions are measured using a pH meter (Metrohm) and found to be ≈2.5 and ≈8.0 for acidic and basic suspensions, respectively. MilliQ water (18.2 MΩ cm at 25° Celsius) was used to dilute the parent suspension to a range of solid volume fractions *ϕ*_0_ ≈ 0.57, 2.8, 5.7 × 10^−5^ for both acidic and basic drops. The corresponding measured pH was approximately 5, 4.5, 4 for acidic suspensions. For the basic suspensions, the calculated pH is in the range 7–7.1 for the three volume fractions considered.

## 3 Results and discussion

**Fig. 2:**
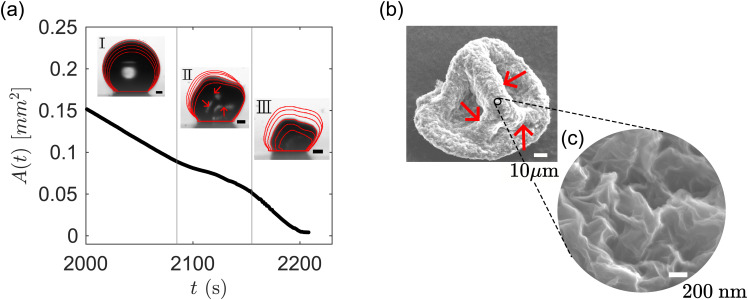
(a) Time evolution of projected drop area *A*(*t*) for *ϕ*_0_ = 5.7 × 10^−5^ and RH = 30%. The insets show the contours of drop edges (red curves) at selected times, superimposed on side view images of the drop. The black scale bars are 50 μm. (b) SEM image of the buckled shell. (c) SEM image at higher magnification of the buckled shell surface shows nano-scale wrinkles.

We start by examining how the drop shape changes in time. Fig. 2 shows the time evolution of the drop's projected area for *ϕ*_0_ ≈ 5.7 × 10^−5^ and pH ≈ 4. In the same figure we also show contours of the drop edges (red curves) at selected times, superimposed on the side images of the drop. From this figure we can identify three characteristic regions. In region I the drop has a nearly spherical shape. Region II begins when the projected drop shape deviates from a circle, approximately at a buckling onset time *t* = *t*_b_ ≈ 2090 s. In region II the rate of change of projected area decreases slightly with respect to region I, because the shape perturbations are growing faster than the evaporation induced change in projected area of the spherical drop. In region III the buckled shell shrinks in volume while preserving its overall shape. Fig. 2b shows the SEM images of the dried buckled capsule considered in Fig. 2a. The side view of the buckled shell is captured by rotating the sample 35° with respect to the angle of incidence of the electron beam, where we can see a correspondence between the folds in Fig. 2b and those in the central panel of Fig. 2a (indicated by red arrows).

While in region III the overall shape does not change, we hypothesize that compression induces small-scale wrinkles, which are visible in Fig. 2c. From the auto-correlation of the intensity profile of the SEM image in Fig. 2c, we estimate a wrinkle wavelength of roughly 100 nm (see Section III in ESI†), one order of magnitude smaller than the length of each GO sheet, and two orders of magnitude larger than the sheet thickness.

### 3.1 Stage I: evaporation

**Fig. 3:**
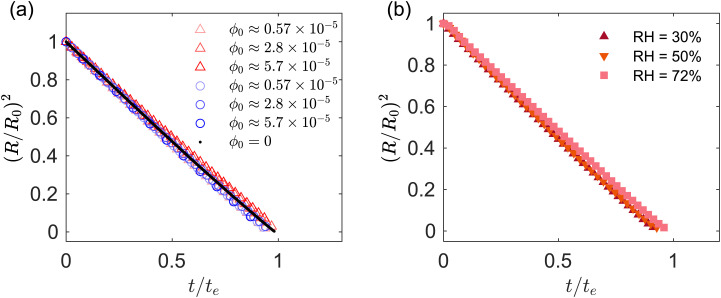
(a) Ratio (*R*/*R*_0_)^2^*vs. t*/*t*_e_. The diameters *R* and *R*_0_ are the current and initial drop radius computed from the projected drop area, respectively 
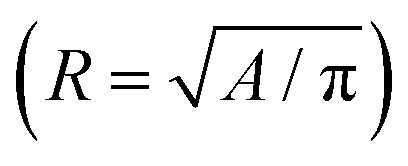
. Red and blue markers correspond to acidic and basic pH, respectively. (b) Ratio (*R*/*R*_0_)^2^*vs. t*/*t*_e_ for different values of relative humidity. The formula used for the time scale *t*_e_ is the same for all the data sets presented. The value of *t*_e_ depends on the value of RH% for the specific experiment considered.

The drop projected area in region I decays linearly in time *t*, following the well-known *d*^2^ law for diffusion-limited evaporation of clean drops.^49^ This is confirmed in Fig. 3a and b, which shows for region I the time evolution of the drop radius squared, plotted against the normalized time *t*/*t*_e_, where *t*_e_ = *ρR*_0_^2^/[*D*(*c*_s_ − *c*_∞_)] is the characteristic evaporation time of a clean water droplet;^19^ here *ρ* is the density of water, *c*_s_ is the saturation vapor concentration and *c*_∞_ is the vapor concentration far away from the drop. Fig. 3a is for fixed RH = 30% and different *ϕ*_0_. Fig. 3b is for different RH and fixed *ϕ*_0_ ≈ 5 × 10^−5^. Fig. 3a compares acidic and basic droplets, while Fig. 3b is for acidic droplets. The experiments were performed for initial drop diameter 2*R*_0_ = 1.5 ± 0.03 mm. Fig. 3 suggests that the effect of the initial particle volume fraction, RH and pH on the evaporation rate is minimal. In region I, the GO drops evaporate approximately following the same law as pure water drops.

As the GO drops evaporate, the particle concentration increases near the air–water interface, so a change in evaporation rate might be expected if some of the particles are adsorbing at the fluid interface. Recent experiments on tracer diffusion across the interface of emulsion drops covered with a monolayer of spherical particles demonstrate that the rate of mass transfer across the interface is unaffected by the presence of the particles at the interface.^50^ Drop evaporation, drop dissolution and tracer diffusion across an interface are driven, from a continuum perspective, by diffusive processes that depend in an identical manner on the difference between the interfacial concentration and the concentration “at infinity”, so similarities between tracer mass transfer at a liquid–liquid interface with spherical particles and evaporation at a water–air interface with sheet-like GO particles may be expected. However, the negligible effect of interfacial particles on the rate of evaporation appears counter-intuitive. After all, if the drop surface was entirely covered with a layer of solid impermeable to diffusion, the evaporation rate would be zero. This apparent paradox can been resolved by analyzing the dependence of the evaporation flux on surface coverage.^51–54^

**Fig. 4:**
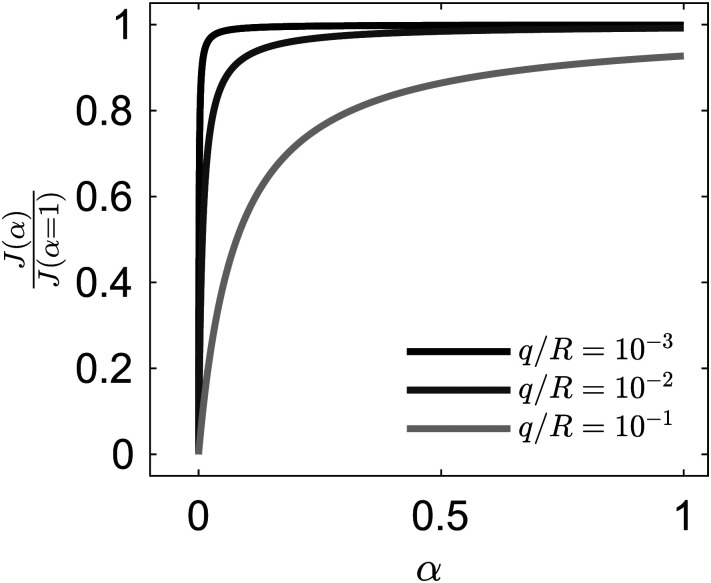
Ratio of vapor mass flux of a particle-covered interface to that of a clean interface, from eqn (1); *q* is the characteristic size of the exposed patch permeable to diffusion and *R* is the drop radius.

The average diffusive mass flux across the surface of a drop covered with particles is approximately the same as the drop surface without particles, provided that (i) a sufficient number of exposed patches of fluid interfaces are homogeneously distributed on the fluid interface and (ii) the size of the exposed patches *q* is small compared to the drop radius (*q*/*R* ≪ 1). This result is suggested by a mathematical model that considers patches of characteristic size *q* homogeneously distributed on the surface of the drop. The patches are regions permeable to diffusion on the impermeable layer made by the GO particle assembly (for a GO monolayer, *q* is a fraction of the particle diameter). On these patches, the vapor concentration is fixed to the saturation concentration *c*_s_. On the impermeable surface of the GO particles, the boundary condition is of homogeneous Neumann type: the gradient of vapor concentration along the normal to the surface is zero. For *t* ≫ *D*/*R*^2^, where *R* is the radius of the drop, the quasi-steady vapor concentration field around the drop satisfies the Laplace equation, ∇^2^*C* = 0.^55^ The mass transfer problem of calculating the mass flux out of the droplet, which corresponds to the solution of ∇^2^*C* = 0 for mixed Neumann–Dirichlet boundary conditions, is mathematically identical to the electrostatic problem of calculating the average electric field on the surface of a dielectric sphere covered with patches held at a fixed electric potential. Asymptotic solutions to the electrostatic problem are available for circular patches located at sufficient distance from each other.^56,57^ The asymptotic expression of Berg & Purcell,^58^ translated in the language of mass diffusion, gives the following ratio between the mass flux when the patches permeable to diffusion cover an area fraction *α* to the maximum flux:1
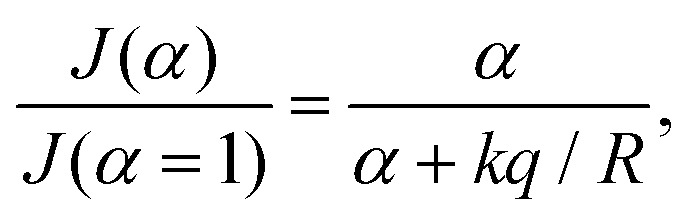
where *k* = π/4. An important insight from this expression is that *α* does not need to be large for *J*(*α*) to be numerically close to *J*(*α* = 1). Indeed, as can be seen in Fig. 4 for selected small values of *q*/*R*, 
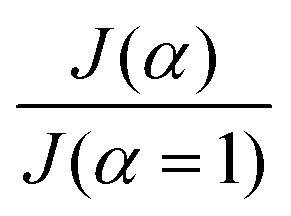
 grows fast with *α*. The linear part of the graph, corresponding to the asymptotic limit 
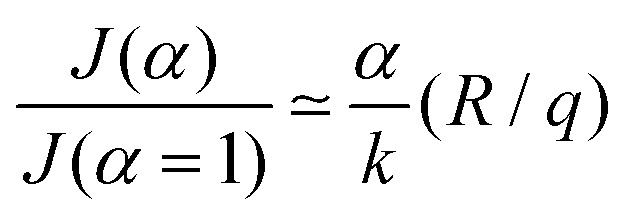
, has a large slope because *R*/*q* is much larger than 1 for all practical conditions encountered in our experiment (taking *q* to be the particle diameter we have *q*/*R* of the order of 10^−3^).

The condition that the patches are homogeneously distributed is essential. To take a limiting case, if the drop was entirely covered with solid with a single exposed fluid interface patch the mass flux would be drastically reduced, because on most of the drop surface the concentration would be significantly smaller than the saturation concentration.

The trend predicted by eqn (1) is qualitative similar to the one predicted by the solution *via* conformal mapping of the 2D Laplace equation for the concentration field in a plane bounded by a periodic flux/no flux line boundary and a line boundary held at a fixed concentration.^53,59^ These planar models, which have been used to explain evaporation from porous media, also predict a strong dependence on *α* but unlike eqn (1) require the prescription of the mass transfer boundary layer thickness. Numerical solutions for diffusion from permeable patches on a sphere have recently become available.^57^

### 3.2 Stage II: onset of buckling

**Fig. 5:**
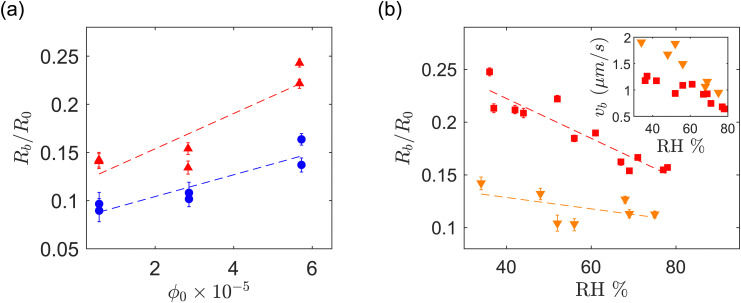
(a) Normalized buckling radius (*R*_b_/*R*_0_) *vs. ϕ*_0_ for different pH ranges. The red triangle marker (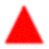) and the blue circle marker (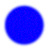) correspond to acidic and basic pH, respectively. The pH of acidic drops corresponding to *ϕ*_0_ ≈ 0.57, 2.8, 5.7 × 10^−5^ are 5, 4.5, 4, respectively. For basic drops the pH is in the range 7–7.1 for all values of *ϕ*_0_ . (b) Normalized buckling radius (*R*_b_/*R*_0_) *vs.* relative humidity RH for drops with acidic pH. The error bar is calculated at the radius *R*(*t*_b_ ± 5 s). The inset shows the velocity of the interface *v*_b_ at the onset of buckling *vs.* RH. The red square marker (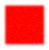) and the yellow triangle marker (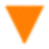) correspond to *ϕ*_0_ ≈ 5 × 10^−5^ and *ϕ*_0_ ≈ 5 × 10^−6^, respectively. The dashed lines serve as a visual guide.

To characterize the onset of buckling in region II we introduce the buckling radius *R*_b_, defined as the minimum drop radius for which *ζ* > 1, where *ζ* = *P*^2^/(4π*A*) is a circularity parameter, with *P* and *A* the projected drop perimeter and area, respectively. Fig. 5a shows *R*_b_, normalized by the initial drop radius *R*_0_, *versus ϕ*_0_ for acidic and basic drops. The buckling radius increases with increasing *ϕ*_0_, a trend that can be explained by the earlier formation of a particle shell for higher initial particle concentration.^1,2^ Indeed, the average mass flux of particles towards the fluid interface is approximately *ϕ*_0_*v*_i_, so for a given fluid interface velocity *v*_i_ the maximum packing fraction is reached earlier if *ϕ*_0_ is larger. Fig. 5b shows *R*_b_/*R*_0_ for acidic drops, for a range of relative humidity values and selected particle volume fractions (in this figure *R*_b_ is found by measuring the deviation from a circular projected contour, see Section IV in ESI†). The inset shows the interface velocity at the onset of buckling *v*_b_. Higher evaporation rate results in higher interfacial velocity and, for a given *ϕ*_0_, packing fraction reaches its maximum earlier.

**Fig. 6:**
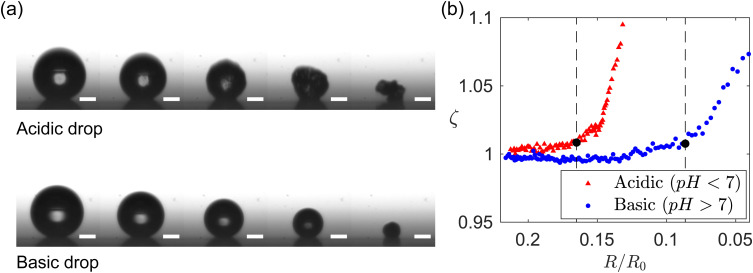
(a) Time evolution of drop morphology comparing acidic and basic drops. The time increases in steps of 25 seconds. Scales bars represent 100 μm. (b) Circularity parameter *ζ* = *P*^2^/(4π*A*) *vs.* normalized radius of the drop. Here *P* and *A* are the projected drop perimeter and area, respectively.

Less trivial is the dependence on pH. For a given *ϕ*_0_, the value of *R*_b_ for acidic drops is about 1.5 times as large as for the basic drops. In Fig. 5 the acidic and basic suspensions are suspensions at different pH ranges obtained from the same manufacturer. To avoid the possible influence of different compositions in the different samples provided by the supplier, we repeat the experiment of Fig. 5 by changing the pH of a suspension of GO from basic to acidic by bubbling CO_2_ through the suspension. The CO_2_ dissolves in water forming carbonic acid, which yield a pH of approximately 4, as measured by a pH meter. The results are shown in Fig. 6a and b. The experimental condition of temperature, humidity and initial drop size are the same as in Fig. 5 with initial particle volume fraction *ϕ*_0_ = 8.5 × 10^−6^. In Fig. 6a we show the time evolution of drop morphology and in Fig. 6b we plot the circularity parameter *ζ vs. R*/*R*_0_ (vertical dashed lines indicate the point of onset of buckling). Fig. 6 confirms that the acidic drop deviates from a spherical shape before the basic drop does. The acidic drop buckles at approximately 2 times larger radius compared to the basic drop. Together, Fig. 5 and 6 provide evidence that the pH has a strong influence on the onset of buckling. A similar observation of pH dependent buckling has been recently reported for dissolving drops laden with spherical colloids.^60^

**Fig. 7:**
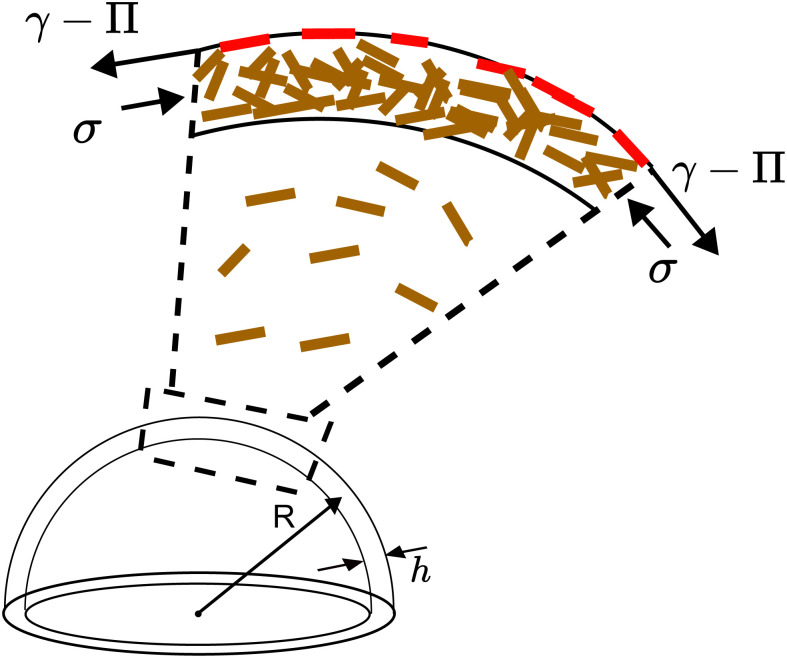
Schematic illustrating the two contributions to the tangential stresses due to GO particles adsorbed at the liquid–air interface (red) and unadsorbed GO particles (brown).

To understand the pH dependence of buckling, we model the particle shell as a particle bi-layer (Fig. 7) composed of (i) the liquid–air interface with adsorbed particles and (ii) the dense layer of completely wetted particles in the vicinity of the liquid–air interface. Clearly, the formation of the layer of adsorbed particles (i) depends on the pH of the drop. Contact between the adsorbed particles yields a surface pressure *Π*;^41,42,61–67^*Π* is the lateral (tangential to the drop surface) force per unit length acting on a line element of particle-laden interface and is positive when the adsorbed particle layer is compressed (provided that the particles touch forming a percolating^67^ or packed layer^42,65,66^). On a scale larger than the particles, the macroscopic tension acting on the fluid interface with the embedded particles is *γ* − *Π*, where *γ* is the bare air–water interfacial tension. The tension on the bilayer must account for the compressive lateral force (per unit thickness) acting on the fully immersed particles (brown particles in Fig. 7). Calling *σ* this tension, the total tension on the bilayer is *γ* − *Π* − *σ*.

For the bilayer to buckle, the bilayer needs to be compressed, so *γ* − *Π* − *σ* ≤ 0. For example, a flat or curved monolayer of particles adsorbed at a fluid interface with *σ* = 0 buckles when *Π* ≃ *γ*, as demonstrated in experiments and numerical simulations.^41,42,63,65–67^ The buckling condition *γ* − *Π* − *σ* ≤ 0 can also be analyzed in terms of fluid pressure. If *p*_i_ is the water pressure inside the drop and *p*_o_ is the air pressure outside the drop, a quasi-static normal force balance on the fluid interface requires^68,69^2
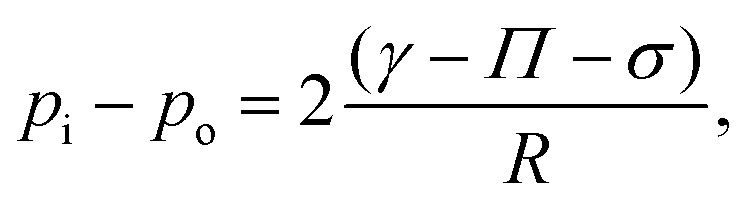
where *R* = *R*_b_. For the drop to buckle there must be a normal force acting on the shell towards the center of the drop. Thus *p*_i_ ≤ *p*_o_, which also gives *γ* − *Π* − *σ* ≤ 0.

For a particle layer having a finite bending rigidity, one may expect that *γ* − *Π* − *σ* should be sufficiently negative for the particle shell to buckle, so the critical condition *γ* − *Π* − *σ* = 0 is approximate. For instance, an Euler beam of Young modulus *E*, moment of inertia *I* and length *L* buckles when the compressive load is larger than π^2^*EI*/*L*^2^. For a homogeneous elastic shell, the critical pressure for buckling is of the order of *p*_c_ ≈ *E*(*h*_b_/*R*_b_)^2^, where *h*_b_ is the shell thickness at the onset of buckling.^70,71^ The maximum pressure from capillary compression is of the order of *γ*/*r*_m_, where *r*_m_ is the characteristic radius of curvature of the small menisci between the interfacial particles.^1,63^ Taking *r*_m_ to be of order of particle size ℓ, we get *γ*/*r*_m_ ∼ 10^5^ Pa, while *p*_c_ is in the range 70–2200 Pa (we take *E* ≈ (*ϕ*_shell_ − 0.002)^2.7^ GPa^44^ and use the values of *h*/*R* measured in Section III C, ESI†). We can thus safely neglect the influence of the bending rigidity and consider the condition *γ* − *Π* − *σ* = 0 a reasonable approximation.

Given that *γ* is independent of pH,^72^ a dependence of the buckling threshold on pH can be explained if either *Π* or *σ* depend on pH. As mentioned in the introduction, there is ample evidence in the literature showing that the main effect of pH at the air–water interface of a GO solution is to change *Π*. Uniaxial compression experiments in a Langmuir trough with an air–water interface populated with a monolayer of GO particles show that the propensity of the GO particles to adhere to the fluid interface is strongly pH dependent.^34^ These experiments show that for acidic solutions (pH = 5.5) the GO particles are adhered to the air–water interface and the monolayer buckles upon compression when *Π* ≈ *γ*.^34,38^ For a basic solutions at pH = 10.5, the GO sheets instead desorb from the fluid interface already at *Π* ≈ 2.5 mN m^−1^. Molecular dynamics simulations show that in an acidic environment the GO particles are attracted to the liquid–air interface while they are repelled from the interface for basic pH.^36^

**Fig. 8:**
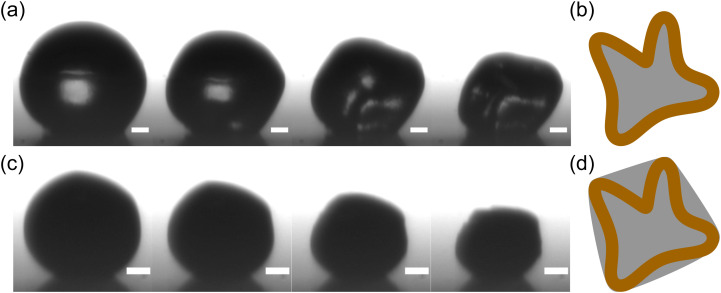
Difference in post-buckling morphology between acidic (a) and basic (c) drops for *ϕ*_0_ ≈ 5.7 × 10^−5^. Concave depressions are observed on the interface of acidic drops, while basic drops exhibit convex interface shapes (better seen in the Movie M1, ESI†). The time increases in steps of 25 s for acidic drops and 10 s for basic drops. Scale bars represent 50 μm. The schematics on the right illustrate the difference in interface shape when particles are adsorbed at the fluid interface (b) or repelled by it (d).

The hypothesis that changes in pH mainly changes *Π* is also supported by the fact that ignoring the effect of pH on *Π* would give a purely convex shape of the fluid interface, as illustrated in the schematic in Fig. 8d, because the absence of adsorbed particles means that the particles can only push outwards on the interface, but not pull inwards. Yet the experimental images clearly show inward indentations of the water–air interface for acidic drops (see Fig. 8a). It may be possible that the contrast of the images for basic drops in Fig. 8b is not sufficient to detect concave depressions. We performed experiments with basic drops at higher concentrations *ϕ*_0_ ≈ 28 × 10^−5^. Since buckling starts earlier for increased *ϕ*_0_, a better contrast is obtained for this set of experiments. The Movie (M1) (ESI†) shows the evaporation of this drop at RH = 30%. The drop has a convex non-spherical shape after the onset of buckling, as suggested in the illustration of Fig. 8d.

### 3.3 Post buckling morphology (after stage III)

**Fig. 9:**
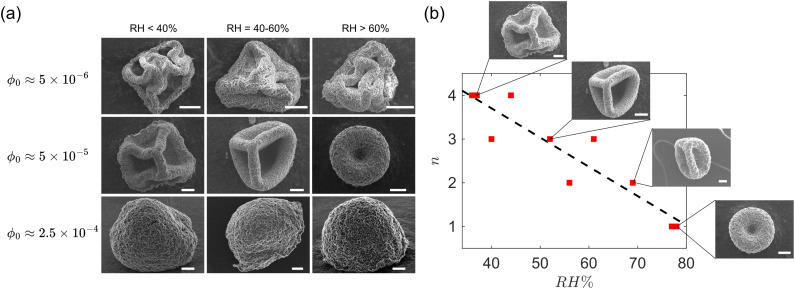
(a) Scanning electron microscope (SEM) images of dried capsules for selected initial concentrations and relative humidity values. (b) Buckling mode number *vs.* relative humidity for *ϕ*_0_ ≈ 5 × 10^−5^. Insets show corresponding SEM images of the buckled capsule. All the scale bars represent 20 μm. Black dashed line serves as a visual guide. All experiments here correspond to GO drops with acidic pH.

We now study the effect of relative humidity and initial particle concentration on the post-buckling morphology of GO drops with acidic pH and initial diameter 2*R*_0_ = 1.65 ± 0.05 mm. Fig. 9a shows SEM images of the dried capsules obtained from acidic drops for a range of values of relative humidity and initial concentrations. For the lowest concentration *ϕ*_0_ ≈ 5 × 10^−6^, buckling leads to highly convoluted folds, while for the highest concentration buckling does not occur. The absence of buckling at *ϕ*_0_ ≈ 2.5 × 10^−4^ suggests the formation of a relatively thick shell. Our observations indicate that for this concentration the drop essentially skips stage II and enters directly stage III where the nearly spherical shell shrinks in volume maintaining a constant shape (see Movie M2 included in the ESI† corresponding to *ϕ*_0_ ≈ 2.5 × 10^−4^ and RH = 32%). We could only detect small deviations from a perfectly spherical shape at *R*/*R*_0_ ≈ 0.34. At this radius, the interface velocity is *v*_i_ = 0.43 μm s^−1^, which gives a particle Péclet number *v*_i_ℓ/*D*_p_ ≈ 0.7. For Péclet number smaller than 1 diffusion could lead to a porous shell of thickness larger than the value estimated by eqn (3), which is a good approximation only for large Péclet numbers. A thick porous shell, formed as a result of the particles assembling into a percolating network, could deform isotropically rather than buckle.

For intermediate values of *ϕ*_0_, we observe a clear correlation between the number of folds in the buckled shape and the value of RH (Fig. 9b). The mode number *n* was taken as the number of concave depressions observed in the SEM images of the buckled capsules. Faster evaporation leads to higher buckling mode numbers. A similar trend and identical buckled shapes have also been reported for evaporating drops laden with spherical colloids.^28^

The observation of reproducible buckled shapes at intermediate *ϕ*_0_ is in sharp contrast to the buckling of elastic shells, investigated in ref. 70, 71 and 73–77, which is known to be sensitive to defects. The repeatable shapes of Fig. 9b suggest a shell with small imperfections of negligible influence. Recent studies on the role of imperfections in shell buckling report that for a small ratio of the size of the imperfection to the shell thickness, the shells become less sensitive to imperfections.^76,77^

The mode number dependence on the evaporation rate (Fig. 9b) could be explained by a geometric argument from perfect shell theory^71^ or by the inertia of the shell.^78,79^ To rule out the second, less plausible hypothesis, knowledge of the thickness of the shell is necessary. Direct measurement of the thickness of the shell in an evaporating drop is challenging, unless sophisticated experimental techniques are used.^29,43^ We estimated the shell thickness by measuring the evaporation of GO suspensions from the tip of a capillary tube placed in a humidity controlled chamber.^80–82^ The water evaporates from the open end of the capillary tube, resulting in the accumulation of the GO particles at the air–water interface and the formation of a planar particle layer. The solid fraction in the layer can be calculated as *ϕ*_s_ ≈ (*v*_c_/*v*_shell_)*ϕ*_0_,^31^ where *v*_c_ is the rate of change of height of the liquid column in the capillary (due to evaporation) and *v*_shell_ is the rate of change of thickness of the particle layer. Detailed explanation of the experiments are found in the ESI.† The measured solid fraction was *ϕ*_shell_ = 0.018 ± 0.004 for the same initial solid fraction and similar range of particle Pe numbers as in the drop experiments of Fig. 9b. The ratio of shell thickness to drop radius at the onset of buckling can be estimated from mass conservation of the particulate phase:^1^3
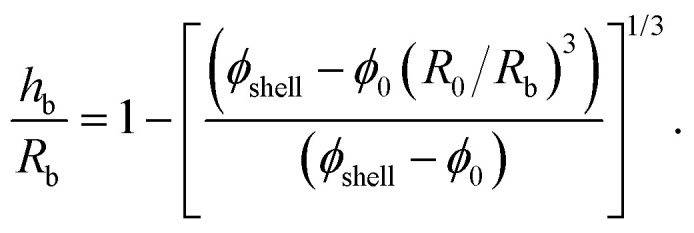
According to this expression, *h*_b_/*R*_b_ decreases with increasing *R*_b_/*R*_0_. Because our experimental data shows that *R*_b_/*R*_0_ increases with decreasing RH (Fig. 5), faster evaporation will be associated to smaller values of *h*_b_/*R*_b_. Using the measured values of *R*_0_/*R*_b_ and *ϕ*_shell_, *h*_b_/*R*_b_ is estimated to be in the range 0.06–0.34, where 0.06 and 0.34 correspond to the smallest and largest values of RH. Thus, the humidity level has a direct effect on the slenderness of the shell at buckling.

The ratio of the kinetic energy of the shell to the bending energy (*B* ∼ *Eh*_b_^3^) of the shell is of the order of *ρ*_shell_*R*_b_^2^*h*_b_*v*_b_^2^/*B*. For buckled GO capsules in Fig. 9, this ratio is in the range 10^−14^ to 10^−10^. Since *ρ*_shell_*R*_b_^2^*h*_b_*v*_b_^2^/*B* ≪ 1, the role of inertia can be safely neglected.

For inertia-less, homogeneous, linear elastic shells, the mode number *n* is obtained by a balance of elastic bending and stretching energy contributions.^70,83^ For a perturbation of amplitude *w* and characteristic wavelength *λ*, the bending energy per unit area is *Eh*^3^(*w*/*λ*^2^)^2^, where *w*/*λ*^2^ is an estimate of the local curvature. The perturbation also results in an in-plane strain *w*/*R* and the corresponding stretching energy per unit area is *Eh*(*w*/*R*)^2^. Balancing the two energies gives a prediction for the mode number *n* ∝ *R*/*λ* ≈ (*R*/*h*)^1/2^. For a constant shell radius, increasing the shell thickness increases the bending energy contribution: it is energetically favorable to select large wavelengths (small mode numbers) as this minimizes the bending energy. For inertia-less, homogeneous, linear elastic spherical shell the mode number is predicted to satisfy^71^

4 *n*(*n* + 1) = [12(1 − *ν*^2^)]^1/2^(*R*_b_/*h*_b_)

**Fig. 10:**
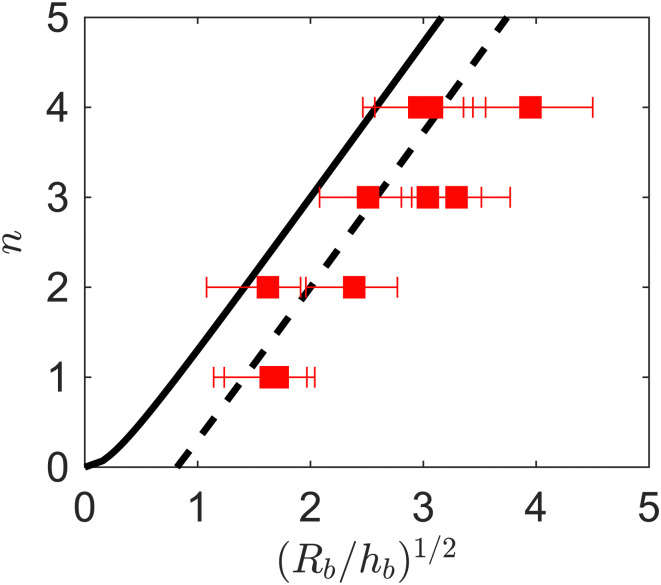
Buckling mode number *n vs.* (*R*_b_/*h*_b_)^1/2^ for *ϕ*_0_ ≈ 5 × 10^−5^. The black curve corresponds to eqn (4). The black dashed line is *n* − 1.

The underlying theory holds for large values of the Föppl-von Kármán number *κ* = *EhR*^2^/*B*;^70,84^ in our case *κ* is in the range 76–2200 for *ϕ*_0_ ≈ 5 × 10^−5^. Fig. 10 shows *n vs.* (*R*_b_/*h*_b_)^1/2^ for *ϕ*_0_ ≈ 5 × 10^−5^. The black curve is the theoretical prediction *n*(*n* + 1) = [12(1 − *ν*^2^)]^1/2^(*R*/*h*)_b_,^71^ where [12(1 − *ν*^2^)]^1/2^ = 3 with *ν* ≈ 0.5 (*ν* is usually taken to be of order 1^44^). It is seen that the prediction follows the experimental trend, but is shifted by a factor of order 1. The prediction does not account for the fact that contact with the substrate reduces the number of concavities. Shifting the mode number prediction by 1 to account for this effect (black dashed line) improves prediction of the experimental data.

### 3.4 Stability of buckled capsules in water

**Fig. 11:**
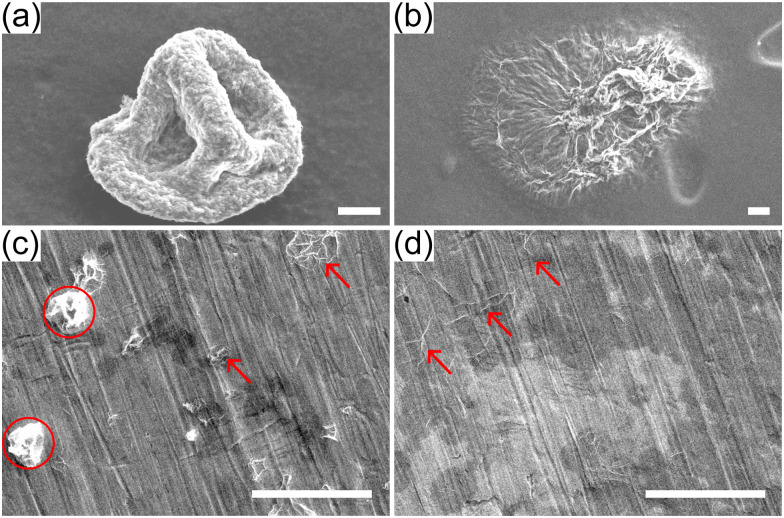
Comparison of dried buckled capsules from single evaporating drops (top panels) and from spray dried drops (bottom panels) after re-suspension in water. Top: SEM images of capsules from acidic (a) and basic (b) drops from experiments on super-hydrophobic substrates. The buckled capsules are re-suspended in water and drop casted onto carbon tapes. Bottom: SEM images of spray-dried GO powders from acidic (c) and basic drops (d). The powders are re-suspended in water and drop casted onto polished copper tapes. Scale bars represent 20 μm.

Fig. 11a and b show the morphology of the dried capsules from drop evaporation experiments of acidic and basic drops for *ϕ*_0_ ≈ 5.7 × 10^−5^. Each dried capsule is first re-suspended in water and then drop casted onto a carbon tape for SEM imaging. The buckled capsule of the acidic drop retains its buckled morphology upon contact with water, while the capsule from the basic drops partially unfolds. A possible explanation is that the thicker shell of basic droplets undergoes a smaller in-plane compression in region III, *A*/*A*_dry_ ≈ 3 as opposed to *A*/*A*_dry_ ≥ 10 for acidic drops. The stability of buckled capsule of graphene oxide re-suspended in water is attributed to the formation of permanent bonds due to plastic deformation of the sheets.^14^ The weaker compaction of basic droplets may not result in the formation of such permanent bonds, so unfolding for basic droplets is more likely.

As an illustration of the practical implications of this observation, powders obtained by spray drying acidic and basic GO suspensions (*ϕ*_0_ ≈ 2 × 10^−3^) at a lab-scale spray dryer are collected and re-suspended in water (see ESI† for experimental details). The suspension is then drop casted onto a polished copper tape and imaged by SEM (Fig. 11c and d). Fig. 11c shows a few buckled capsules (red circles) and some unfolded capsules (red arrows). Fig. 11d shows only flat sheets with some folds, indicating that all buckled capsules have unfolded. Needless to say, the conditions in a spray drier are much more complicated than in our single-drop experiments, but the simple test of Fig. 11c and d illustrates that the pH of the solution has a drastic effect on the ability of GO capsules produced by spray-drying to maintain their shape when redispersed in water.

## 4 Conclusions

We investigated experimentally the evaporation of water drops containing graphene oxide nanosheets. Single drops are placed on a superhydrophobic surface and are observed from the side by a camera. The buckled capsules are imaged by SEM. Different stages of evaporation are identified, and the effects of solid concentration, pH and relative humidity are assessed. Evaporation gives rise to a particle-rich shell that buckles, forming folded structures. For certain ranges of particle concentration and relative humidity the folded structures display distinct buckling modes (Fig. 9).

Before buckling, when the drop has a nearly spherical shape, the evaporation rate is found to be independent of the initial particle concentration. A theoretical model based on Berg & Purcell's^58^ asymptotic solution for a sphere covered with a uniform distribution of patches permeable to the evaporation flux is used to demonstrate that even in the presence of GO particles adsorbed on the fluid interface, the evaporation flux across the particle-laden and clean drop surface are almost identical.

The initial particle concentration and pH have a marked effect on the drop radius at which buckling occurs. Buckling of basic droplets (pH > 7) occurs later, for a given particle concentration, than buckling of acidic drops. Dried capsules formed from acidic drops do not unfold when resuspended in water, unlike capsules from basic drops. There is therefore a link between onset of buckling time and mechanical stability in water of the produced capsules.

The observation of the shape of the fluid interface post buckling seems to suggest that the pH dependent adsorption of GO particles to the air–water interface plays an important role in, and possibly controls, the onset of buckling. Our interpretation of the condition for the onset of buckling, based on particle adsorption, is different from the one assumed by many published models for spherical colloids, which consider that only the particles completely immersed in the fluid are responsible for buckling.^1,29^

For a range of intermediate concentrations and relative humidities, very controlled buckled structures could be obtained. Analysis of the buckling modes of these structures indicate that the particle shell buckles essentially like a inertialess deflated elastic shell. The repeatability in which the interface buckles in our experiments to yield such gallery of shapes is remarkable, since buckling is typically considered to be an instability strongly sensitive to perturbations. The shape of deflated elastic shells is notoriously dependent on the presence of defects. In our experiments, inhomogeneities in the particle distribution, if present, do not seem to affect the overall shape of the capsules.

We report an increase in mode number (number of depressions) for increasing evaporation rate, see Fig. 9b. This trend can be explained by the dependence of the mode number *n* on the slenderness of the shell at buckling, as measured by *h*_b_/*R*_b_. The mode number increases with *R*_b_/*h*_b_, approximately as ∼(*R*_b_/*h*_b_)^1/2^ (eqn (4)), and *R*_b_/*h*_b_ in turn depends on the humidity level, as demonstrated by the data in Fig. 5.

Spray drying of acidic drops may be useful to obtain stable suspensions of GO capsules, as we demonstrate in Fig. 11. The emergence of hierarchical folds due to buckling – we report undulations ∼100 μm accompanied by small scale undulations of the order of 100 nm – could be exploited, for example, to produce hierarchical particulate materials.^7,85^

## Data availability

The datasets generated and analyzed in this study are available at the 4TU.ResearchData repository at: https://doi.org/10.4121/d82a8328-7062-43b8-882c-42f28cc4251b.

## Conflicts of interest

There are no conflicts to declare.

## Supplementary Material

SM-021-D4SM01342E-s001

SM-021-D4SM01342E-s002

SM-021-D4SM01342E-s003
