# Supplementary material for: Evaporation driven buckling of a drop laden with graphene oxide nanosheets
Source: Soft Matter. 2025 Feb 17;21(13):2518–28. doi: 10.1039/d4sm01342e (PMC11892344; doi:10.1039/d4sm01342e)
Supplement: SM-021-D4SM01342E-s003 [file SM-021-D4SM01342E-s003.pdf]

Supplementary Information (SI) for Soft Matter.
This journal is © The Royal Society of Chemistry 2025

Soft Matter

ARTICLE TYPE

Supplementary information: Evaporation driven buckling of a drop laden with graphene oxide nanosheets

Suriya Prakash,[a] Eva Krolis,[b] Alvaro Marin,[b] and Lorenzo Botto.[a‡]

1 Drop contact angle during evaporation

The time evolution of the contact angle of a dilute ($\phi_0 \approx 0.57 \times 10^{-5}$) GO drop during evaporation is shown in figure 1. The contact angle is approximately 155° for most of the drop evaporation time, implying evaporation in a constant contact angle mode.

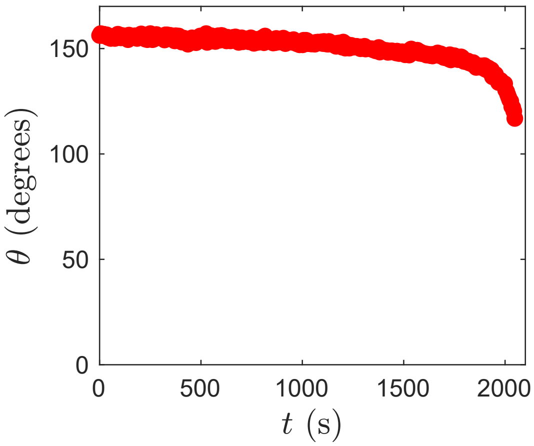

Fig. 1 Time evolution of contact angle of a GO drop during evaporation.

2 GO sheet length and thickness estimation

To estimate the GO thickness we used atomic force microscopy (AFM). A GO suspension with initial particle concentration $\phi_0 \approx 5 \times 10^{-7}$ is drop casted onto a silicon wafer with a 300 *nm* coating of SiO_2 (PI-KEM). Prior to drop casting the silicon wafer is cleaned with acetone, IPA, ethanol and subject to sonication in an ultrasonic bath with Milli-Q water. A region of 20×20 $(\mu m)^2$ is imaged in the AFM using tapping mode. The data is processed with the open-source software Gwyddion to obtain the height map shown in Fig. 2. The thickness statistics are obtained by a in-house developed MATLAB code. The measured average sheet thickness is $t = 1.00 \pm 0.14$ nm. The lateral sizes were measured using scanning electron microscopy images of the same silicon wafer. The average sheet length is $\ell = 1.08 \pm 0.44$ μm.

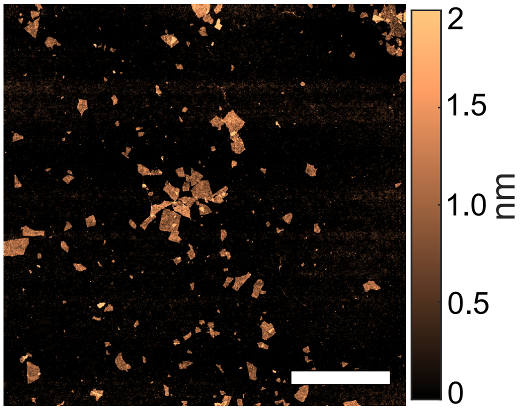

Fig. 2 AFM image of the GO sheets drop casted on silicon wafer. The color map corresponds to the height data. The white scale bar represents 5 μm.

3 Wavelength of small scale wrinkles

To estimate the wavelength of small scale wrinkles in the SEM image of the buckled aggregate in Fig. 2 c, we compute the autocorrelation of the intensity profile. The intensity autocorrelation can be an approximation of the height-height correlation of the small scale wrinkles in the buckled capsule. The average autocorrelation function becomes zero approximately for $\tilde{x} \simeq 100$ *nm*. We take this to be the typical wavelength of the small scale wrinkles.

4 Radius at the onset of buckling

In Fig. 5 (a) in the main text, the radius at the onset of buckling is found by measuring the deviation of the projected contour of the drop from a circle by using a circularity parameter ζ. In Fig. 5 (b) we introduce a new parameter Φ, the difference in circumference of a fitted ellipse to the drop contour and the measured circumference of the drop contour from the image of the drop. Figure 4 shows Φ vs. time (in seconds) for a typical GO drop during evaporation. As the drop undergoes buckling there is a sharp increase in Φ which we identify as the onset of buckling time t_b and the corresponding radius R_b.

[a] *Department of Process & Energy, Faculty of Mechanical Engineering, TU Delft, The Netherlands.*
[b] *Physics of fluids, Faculty of science & technology, University of Twente, Enschede, The Netherlands.*
‡ Email: L.botto@tudelft.nl

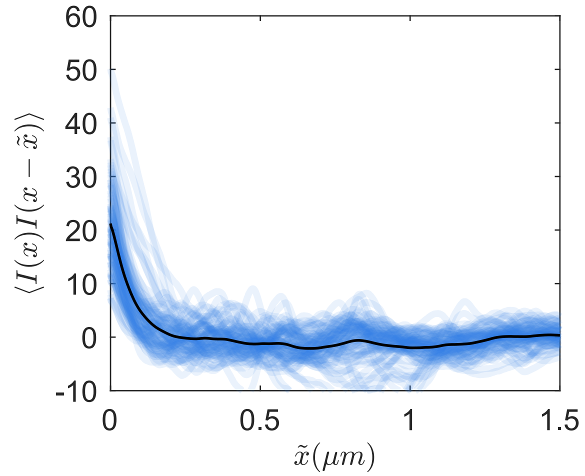

Fig. 3 Autocorrelation function of the intensity profile from the SEM image of Fig. 2 c. The blue curves indicate the autocorrelation function along each row of pixels and the black curve represents the average over all rows.

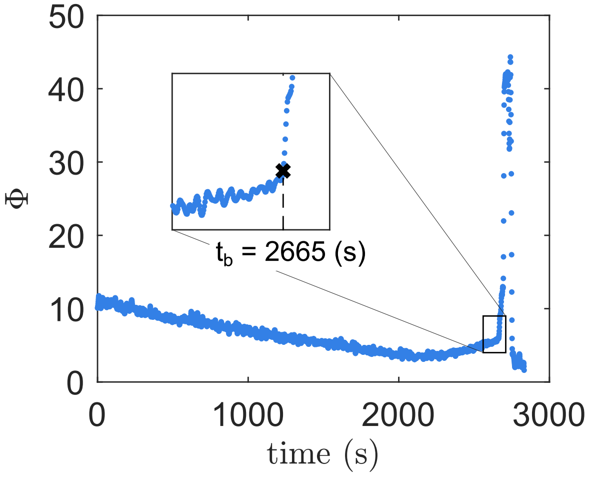

Fig. 4 Difference (Φ) between the circumference of a fitted ellipse and the circumference measured from the side view image of the drop. Inset shows a sharp increase in Φ for time $t = t_b \approx 2665$ s.

5 Capillary evaporation experiments

Figure 5 a shows the schematic of the experimental setup (not to scale) to measure the shell volume fraction. A glass capillary tube with internal diameter 0.4 mm is held perpendicular to gravity, with its open end exposed to a humidity controlled chamber. The other end of the tube is attached to a water reservoir to maintain a constant pressure head[1]. The open end of the capillary tube is filled with the GO suspension and the evaporating GO suspension is separated from the water in the reservoir by an air bubble that is trapped between them. The air bubble saturated with water vapour ensures that the GO suspension evaporates only from the open end of the capillary tube.

The height of water in the reservoir remains unchanged during evaporation, as the reservoir diameter is much larger than diameter of capillary tube, maintaining a constant pressure $P_A > P_{atm}$ between the reservoir and the open end of the capillary tube. The higher pressure (P_A) prevents the GO suspension from retracting into the capillary tube from the open end during evaporation (see Fig. 5 b, where the system is imaged by a camera with a large field of view). The higher pressure also renders the liquid-air interface, at the open end of the capillary, planar. Another camera with zoom lens records the open end of the capillary to visualize the growth of the particle dense layer (see Fig. 5 c). Since the capillary tube is held horizontal, the shell formation is not affected by sedimentation. Both the camera are synchronized to capture images at the same instance at 0.05 fps.

The height of the liquid column in the capillary can be found by balancing the mass loss and the evaporation flux. The height of the liquid column H is[2]

$$H(t) = H_0 - \frac{4D(c_s - c_\infty)t}{\pi \rho_\ell r_i} \quad (1)$$

Here ρ_ℓ is the liquid density, H_0 is the initial height of the liquid column, c_s is the saturation vapour concentration, c_∞ is the vapour concentration far away from the open end of the capillary and r_i is the internal radius of the capillary. Figure 6 shows the normalized liquid column height as a function of time for GO suspension with initial concentration $\phi_0 \approx 5.7 \times 10^{-5}$. The experiments are performed with initial height $H_0 \approx 40$ mm and RH $= 32$ and 60%. The dashed lines are theoretical predictions from Eqn. 1. It can be seen that the experimental data agree well with the linear trend predicted by Eqn. 1 until $t \approx 6000$ s and $t \approx 15000$ s for $RH = 32$ and 60%, respectively. A slowing down of the evaporation rate occurs for longer times. Possible causes of deviation from Eqn. 1 are the recession of the fluid interface into the dense particle layer or the dependence of pressure on the microscopic meniscus curvature[3]. From Fig. 6 we calculate the rate of change of height of the liquid column $v_c = 2, 1.26$ μm/s corresponding to RH $= 30, 60$%, respectively.

To quantify the thickness of the dense particle layer, we use the image data from the camera with a zoom lens (see Fig. 5 c). Figure 7 a shows the open end of the capillary tube with a dense particle layer. The region where the light intensity is measured indicated by a green box which is located just above the tip of the capillary tube. The intensity is averaged in the y direction and

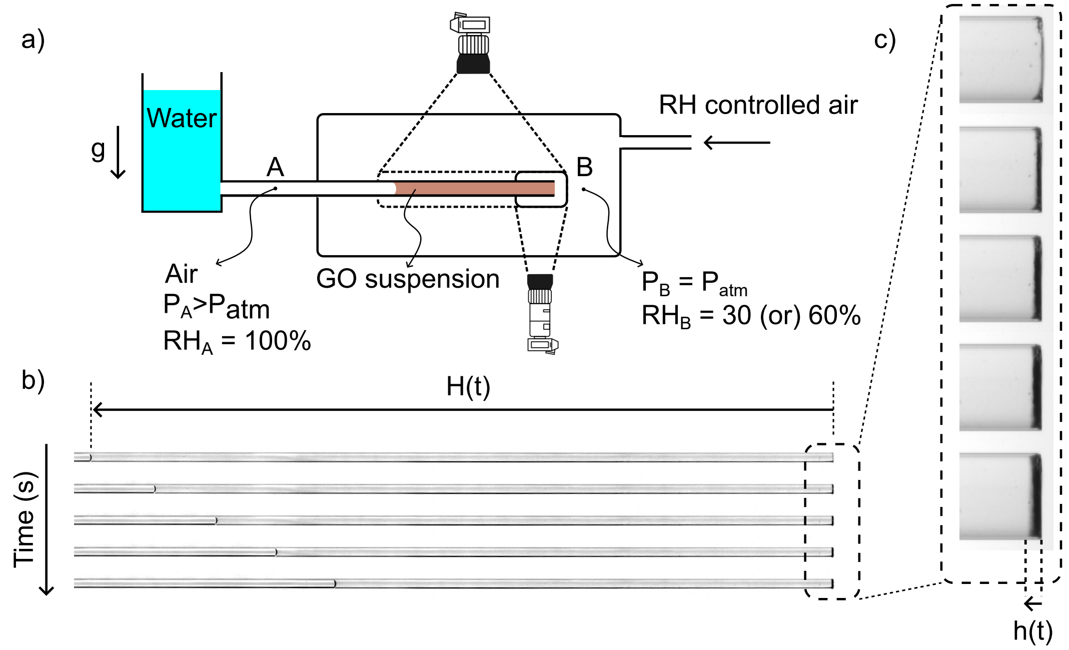

Fig. 5 a) Schematic of the setup for studying the evaporation of GO suspension from a capillary tube. b) Images from the camera with a large field of view shows the height $H(t)$ of the liquid column of the GO suspension. c) Images from the camera with a zoom lens show the shell layer height $h(t)$.

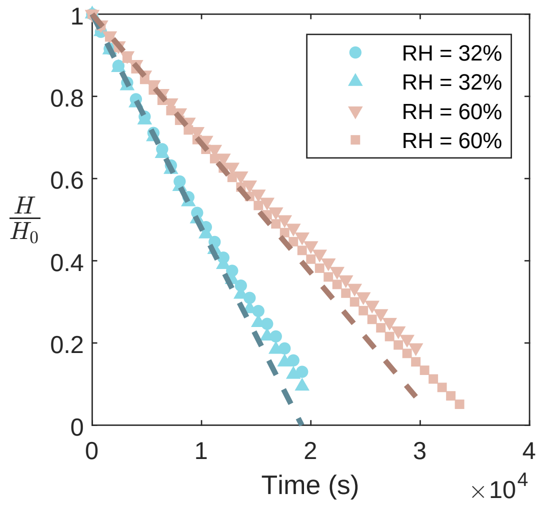

Fig. 6 Height of the liquid in the capillary normalized by initial height vs. time for two humidity conditions. The initial GO concentration is $\phi_0 \approx 5.7 \times 10^{-5}$. The dashed lines are theoretical predictions from Eqn. 1.

plotted as a function of x distance in Fig. 7 b. The intensity value changes from approximately 50 to 200, corresponding to the transition from the dense particle layer to the dilute GO suspension. The intensity curves have a sigmoidal shape and it translates in the x direction as time progresses. By finding the displacement of the sigmoid with respect to time we compute the velocity of growth of dense particle layer. Figure 7 c is a a space-time plot of the intensity over time. The contour line corresponding to the intensity values of 70 visually separates the dense particle layer and the dilute GO suspension. We observe that the dense layer height h scales proportional to $\sqrt{t}$ for long times. In Ref.[3] such dependence was predicted for times for which the fluid interface receded into the particle layer, giving rise to an evaporative mass flux that decreases with increasing h. The growth velocity of the dense particle layer v_{shell} is calculated by a linear fit in the time range where the evaporation rates agree well with the linear diffusive behavior expressed by Eqn. 1 (see Fig. 6). From mass balance, the packing fraction of the GO at the dense particle layer is $\phi_{shell} \approx (v_c/v_{shell})\phi_0$[4]. This expression gives $\phi_{shell} = 0.018 \pm 0.004$. The standard deviation is obtained by choosing different values of intensities (70 - 180) to be the threshold for shell layer.

6 Spray drying experiments

The spray drying experiments are performed in a lab scale spray dryer (Buchi S-300). The initial volume fraction is 0.002 for both acidic and basic suspensions. The suspension is atomized into fine drops of typical radius ≈ 20 μm by a compressed nitrogen at 6 Bars in a spray nozzle. The drops are dried by hot air in a co-flow configuration. The drying air is at 200° Celsius and the air mass flow rate is 30 m^3/hr. Experimental conditions are identical for acidic and basic drops.

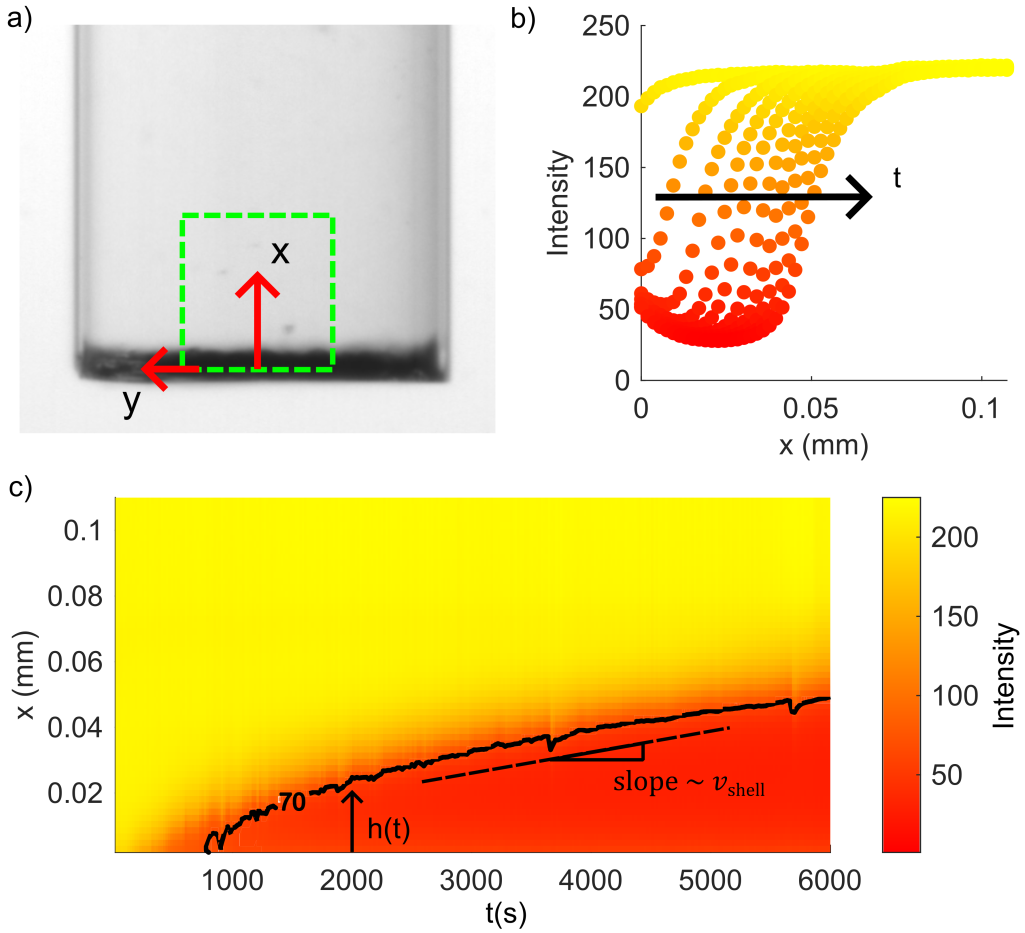

Fig. 7 a) Image of the open end of the evaporating capillary tube. The green box indicates the area in which light intensity is measured. b) Light intensity in the green box (averaged in the y direction) is plotted against x distance. The arrow indicates the progression in time t. c) Space time plot of the average intensity in the green box over time. The black curve is the contour line corresponding to intensity value 70. The slope of the contour line corresponds to velocity of growth of the dense particle layer v_{shell}.

References

1 K. Roger and J. J. Crassous, *Proceedings of the National Academy of Sciences*, 2021, **118**, e2105530118.

2 L. T. Raju, C. Diddens, J. Rodríguez-Rodríguez, M. N. Van Der Linden, X. Zhang, D. Lohse and U. Sen, *Journal of Fluid Mechanics*, 2024, **983**, A21.

3 H. Pingulkar and J.-B. Salmon, *Soft Matter*, 2023, **19**, 2176–2185.

4 E. Guazzelli and J. F. Morris, *A physical introduction to suspension dynamics*, Cambridge University Press, 2011, vol. 45.
